# Acute kidney injury in septua- and octogenarians after cardiac surgery

**DOI:** 10.1186/1471-2261-11-52

**Published:** 2011-08-11

**Authors:** Michael Ried, Thomas Puehler, Assad Haneya, Christof Schmid, Claudius Diez

**Affiliations:** 1 University Medical Center Regensburg, Department of Cardiothoracic Surgery, Franz-Josef-Strauß-Allee 11, 93049 Regensburg, Germany

**Keywords:** Acute kidney injury, cardiac surgery, extracorporeal circulation, mortality, septuagenarians, octogenarians

## Abstract

**Background:**

An increasing number of septua- and octogenarians undergo cardiac surgery. Acute kidney injury (AKI) still is a frequent complication after surgery. We examined the incidence of AKI and its impact on 30-day mortality.

**Methods:**

A retrospective study between 01/2006 and 08/2009 with 299 octogenarians, who were matched for gender and surgical procedure to 299 septuagenarians at a university hospital. Primary endpoint was AKI after surgery as proposed by the RIFLE definition (Risk, Injury, Failure, Loss, End-stage kidney disease). Secondary endpoint was 30-day mortality. Perioperative mortality was predicted with the logistic European System for Cardiac Operative Risk Evaluation (EuroSCORE).

**Results:**

Octogenarians significantly had a mean higher logistic EuroSCORE compared to septuagenarians (13.2% versus 8.5%; p < 0.001) and a higher proportion of patients with an estimated glomerular filtration rate (eGFR) < 60 ml × min^-1 ^× 1.73 m^-2^. In contrast, septuagenarians showed a slightly higher median body mass index (28 kg × m^-2 ^versus 26 kg × m^-2^) and were more frequently active smoker at time of surgery (6.4% versus 1.6%, p < 0.001). Acute kidney injury and failure developed in 21.7% of septuagenarians and in 21.4% of octogenarians, whereas more than 30% of patients were at risk for AKI (30% and 36.3%, respectively). Greater degrees of AKI were associated with a stepwise increase in risk for death, renal replacement therapy and prolonged stays at the intensive care unit and at the hospital in both age groups, but without differences between them. Overall 30-day mortality was 6% in septuagenarians and 7.7% in octogenarians (p = 0.52).

The RIFLE classification provided accurate risk assessment for 30-day mortality and fair discriminatory power.

**Conclusions:**

The RIFLE criteria allow identifying patients with AKI after cardiac surgery. The high incidence of AKI in septua- and octogenarians after cardiac surgery should prompt the use of RIFLE criteria to identify patients at risk and should stimulate institutional measures that target AKI as a quality improvement initiative for patients at advanced age.

## Background

Recent estimates showed that significant proportions of the United States and Western European population are greater than 65 years of age [1] and the elderly also represent the fastest growing age group in the Western world [2]. In 2009 in Germany, 50.8% of patients undergoing cardiac surgery with cardiopulmonary bypass were older than 69 years and 11.8% older than 80 years [3]. The number of patients > 70 years with preoperative chronic kidney disease steadily increased and acute kidney injury (AKI) still remains a frequent complication in up to 30% of patients after cardiac surgery [4]. In particular, the elderly are more susceptible to developing AKI, not only because of the physiological and anatomical changes in the ageing kidney, but also because of concomitant diseases and medications, e.g. nonsteroidal antinflammatory drugs [5]. A Spanish study showed that the incidence of AKI in the general population was 3.5 times higher in patients > 70 years [6] and even 5 times higher in octogenarians [7].

Preoperative renal impairment is independently associated with increased short- and long-term mortality after cardiac surgery [8-10]. Even small increases of serum creatinine significantly impact the outcome [11].

We examined in this study the incidence of AKI in septua- and octogenarians and the impact of AKI on 30-day mortality after cardiac surgery.

## Methods

### Patients & study design

We retrospectively studied 598 patients ≥70 years, who underwent elective bypass, valve or combined bypass and valve surgery with cardiopulmonary bypass (CPB) support between 01/2006 and 08/2009 at the University Medical Centre Regensburg. The study was approved by the local ethics committee, but individual consent was waived because of the study's retrospective design and data collection from routine care.

During the study period, 299 octogenarians (age ≥80 years) were operated on with CPB. This subgroup was matched for gender and operative procedure with 299 septuagenarians (age 70 - 79 years), who underwent cardiac surgery during the same period. Patients with preoperative renal replacement therapy (RRT) were excluded.

Preoperative risk evaluation and variable definition was done with the European System for Cardiac Operative Risk Evaluation (logistic EuroSCORE) [12]. Serum creatinine (SCr) was measured (in μmol/L and converted to mg/dL; 1 mg/dL = 88.4 μmol/L) at the day of hospital admission and glomerular filtration rate (GFR) was estimated with the abbreviated Modification of Diet in Renal Disease (MDRD) equation [13] and expressed in mL/min/1.73 m^2^. SCr was also measured at following time points: arrival at the intensive care unit, 6, 12, 24, 48 and 72 hours after surgery. In addition, SCr was routinely determined one day before discharge. Postoperative AKI was defined according to the RIFLE criteria (Risk (increase in SCr > 1.5x), Injury (increase in SCr > 2.0x), Failure (increase in SCr > 3.0x), Loss, End-stage kidney disease) [14]. The highest SCr value within 72 hours after surgery was used to calculate the relative change to baseline SCr before surgery. Baseline SCr was defined as the serum creatinine at the day of hospital admission, usually one day before surgery. Thus, the RIFLE score in our analysis is basically an assessment of the highest creatinine up to 72 hours after surgery. Oliguria was defined as an urine output < 400 mL/24 h.

Primary endpoint of the study was the incidence of new-onset AKI within 72 hours after surgery. Secondary endpoint was 30-day mortality.

### Operative techniques

All operations were performed with either full or partial upper sternotomy and under conventional CPB. Minimized CPB was not used to prevent possible bias. The ascending aorta and the right atrium were cannulated for bypass and aortic valve surgery, whereas bicaval cannulation was used for mitral valve procedures. The selection of valve prosthesis was based on patient's preference. Cardiac anesthesia was performed according the institution's guidelines.

### Statistical analysis

Stata 10.1 SE (StataCorp., College Station, TX, USA) were used for statistical analysis. Continuous variables were first tested for normality by Q-Q-plots and described with either mean with standard deviation (SD) or, if non-normally distributed, with median and interquartile range (IQR). Comparison of normally-distributed continuous variables was done with Student's t-test or with Mann-Whitney's test, if non-normally distributed.

Categorical data were presented as frequencies. Fisher's exact test was used for data in a 2 × 2 table and chi square test for n × k tables. Univariate logistic regression was used to determine the association between AKI as defined by RIFLE and 30-day mortality. Goodness of fit was tested with the Hosmer-Lemeshow test and discriminatory performance with receiver operator analysis (ROC). A p-value < 0.05 was considered significant.

## Results

### Demographic data

Demographic data were summarized in Table 1. Octogenarians had a significantly higher preoperative logistic EuroSCORE (13.2% versus 8.5%; p < 0.001), which is mainly based on the advanced age in the score calculation. In addition, they had a slightly higher preoperative serum creatinine and more frequently an eGFR < 60 ml × min^-1 ^× 1.73 m^-2 ^reflecting a higher proportion of chronic kidney disease stages III to V in patients ≥ 80 years. In contrast, significantly more septuagenarians were active smokers at time of surgery and had a higher body mass index.

**Table 1:** Demographic data and surgical procedures

Variable	Septuagenarians (n = 299)	Octogenarians (n = 299)	p-value
Age [years]	74 ± 2.8	82 ± 2.1	< 0.001
Female [%]	46	46	1.00
Body mass index [kg × m^-2^]^B^	28 (25; 31)	26 (24; 28)	< 0.001
COPD [%]	8.7	8.4	0.89
Atrial fibrillation [%]	13.4	14.4	0.81
Insulin-dependent diabetes [%]	11	8.4	0.33
Ejection fraction [%]	61 ± 13	59 ± 13	0.34
Logistic EuroSCORE [%]^A^	8.5 (7.7 to 9.4)	13.2 (12.1 to 14.4)	< 0.001
Recent myocardial infarction [%]	24.1	28.4	0.27
Serum creatinine on admission [mg/dL]^B^	1 (0.8; 1.2)	1.1 (0.9; 1.3)	0.05
eGFR < 60 ml × min^-1 ^× 1.73 m^-2 ^[%]	34.4	44.0	0.02
Active smoker [%]	6.4	1.6	0.006
Previous cardiac surgery [%]	7.9	7.9	1.00
Isolated bypass surgery [%]	41.1	41.1	1.00
Isolated valve surgery [%]	33.1	33.1	1.00
Combined valve and bypass surgery [%]	25.8	25.8	1.00

^A ^- Shown with 95% confidence interval

^B ^- Data shown as median with interquartile range

### Perioperative data

Perioperative data were shown in Table 2. Apart from a slightly reduced, clinically insignificant, aortic cross clamp time in octogenarians and a higher, but insignificant, frequency of rethoracotomy in septuagenarians, there were no significant differences between both groups. Patients with isolated CABG had a left internal mammary artery (LIMA) use of 89% (septuagenarians) and 86% (octogenarians) without statistical difference. The frequency of left anterior descending artery (LAD) disease was 95% and 94%, respectively. Patients with combined procedures had a less frequent use of left internal mammary artery (71% in septuagenarians versus 64% in octogenarians; p = 0.39). In those patients there was also a lower prevalence of left anterior descending artery disease.

**Table 2:** Perioperative data

Variable	Septuagenarians (n = 299)	Octogenarians (n = 299)	p-value
Aortic cross-clamp time [min]	60 (46; 80)	57 (43; 75)	0.02
ICU-stay [days]	1 (1; 4)	2 (1; 4)	0.87
Hospital stay [days]	12 (10; 16)	13 (10; 17)	0.24
Respiratory failure [%]^A^	8.4	9.0	0.89
Ventilation time [hours]	12 (9; 18)	12 (9; 19)	0.25
Re-Intubation [%]	6	7.7	0.52
Temporary tracheotomy [%]	2.7	3.3	0.81
Central neurological event [%]^B^	2.7	5.4	0.14
Redo-Thoracotomy [%]	10	5.4	0.05
Drain loss [mL]	500 (300; 800)	450 (300; 750)	0.18
Use of left internal mammary artery [%]^C^	89	86	0.85
Use of left internal mammary artery [%]^D^	71	64	0.39
Number diseased vessels^D^	2	3	0.21
Number of grafts [n]	2 (2; 3)	3 (3; 2)	0.67
Low cardiac output syndrome [%]	2	3	0.60

^A ^- Decrease pO_2 _≤50 mmHg ± increase pCO_2 _> 60 mmHg, tachypnea > 40/min.

^B ^- Including transient/prolonged ischemic neurological deficit and stroke.

^C ^- Only in patients with isolated bypass and combined valve/bypass surgery.

^D ^- Only patients with combined valve/coronary surgery.

^E ^- Data shown as median.

### Postoperative renal function

The RIFLE criteria were used to characterize postoperative acute kidney injury among the study sample (Table 3). The overall incidence of AKI (RIFLE stages 'injury' plus failure') was observed in 21.0% (n = 53) of septuagenarians and in 21.4% (n = 64) of octogenarians. Every fifth patient in both groups developed an acute kidney injury (RIFLE stage "Injury", 16.9% versus 16.4%, p = 1.00) or an acute renal failure (RIFLE stage "Failure", 4.8% versus 5%, p = 0.98). Significant differences, however, could not be observed between both groups. Almost 30% (n = 87) of Septuagenarians and 36.3% (n = 109) of octogenarians were classified as "Risk" (p = 0.07).

**Table 3:** RIFLE classification of postoperative acute renal injury

Septuagenarians (n = 299)			Octogenarians (n = 299)	
**Stage**	**n; %**	**30-Day mortality (n; %)**	**n; %**	**30-Day mortality (n; %)**

No AKI	149; 49.6%	4; 2.7%	126; 42.3%	6; 4.8%
Risk	87; 29.2%	5; 5.7%	109; 36.3%	6; 5.5%
Injury	49; 16.9%	5; 10.2%	49; 16.4%	5; 10.2%
Failure	14; 4.8%	4; 28.6%	15; 5.0%	6; 40%

**Figure 1:**
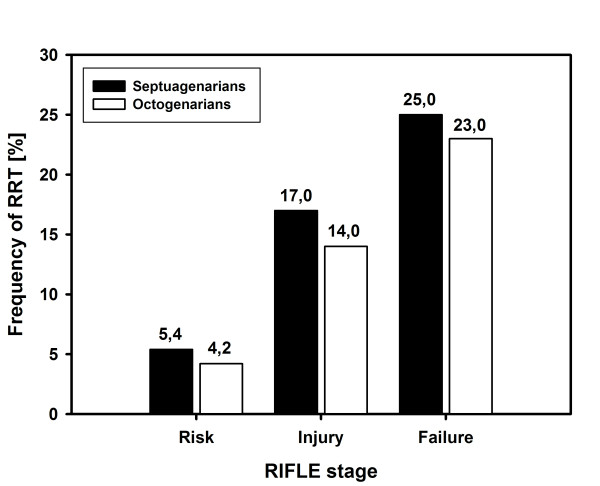
**Bar graph depicting the stepwise in increase (in %) of renal replacement therapy (RRT) according to advanced RIFLE stages in septua- and octogenarians**. There were no significant differences between both groups at the indicated RIFLES stages.

Postoperative temporary renal replacement therapy (RRT) was necessary in 20/299 (6.7%) of septuagenarians and 16/299 (5.4%) of octogenarians (p = 0.60). There was a stepwise increase in the frequency of RRT from RIFLE stage "risk" to "failure" (Figure 1), but without significant differences between both groups. The median time for RRT initiation was 58 hours (47; 69) after surgery. Renal replacement therapy was indicated in nine patients (9/196, 4.8%; 5 septuagenarians and 4 octogenarians) in RIFLE stage "at risk", in 15 patients at RIFLE stage "Injury" (15/98, 15.5%; 8 septuagenarians and 7 octogenarians) and in seven patients (7/29, 24%; 3 septuagenarians and 4 octogenarians) in RIFLE stage "failure".

The indications for RRT also did not differ between both groups and were as follows (stratified to RIFLE stage): Risk: severe metabolic, i.e. lactic, acidosis (n = 3), oliguria (n = 6); Injury: metabolic acidosis (n = 2), severe hyperkalemia (K^+ ^> 6.8; n = 1), oliguria (n = 12); Failure: metabolic acidosis (n = 1), oliguria (n = 6).

The incidence of kidney injury after surgery stratified to procedure is shown in Table 4. Irrespective of age group, combined valve and coronary surgery had the highest incidence of renal failure (each 9%).

**Table 4:** RIFLE classification of postoperative acute renal injury stratified to type of surgery

	Septuagenarians (n = 299)				Octogenarians (n = 299)			
	No AKI [%]	Risk [%]	Injury [%]	Failure [%]	No AKI [%]	Risk [%]	Injury [%]	Failure [%]
CABG	56	25	16	2	37	37	24	3
Valve	45	31	20	4	48	36	11	4
Combination	46	33	14	9	44	35	13	9

Different AKI stages were associated with increased 30-day mortality (Table 3) and risk of mortality increased among patients with greater degree of AKI. The highest observed mortality was in patients with RIFLE stage "Failure", where 28.6% (n = 4/14) of septuagenarians and 6/15 (40%) octogenarians died (p = 0.51). In both age groups were 10.2% non-survivors (5/49) at RIFLE stage "Injury" (p = 1.00) and almost 6% of patients at RIFLE stage "Risk" did not survive (p = 0.94). Overall observed 30-day mortality was 6% in septuagenarians (18/299) and 7.7% in octogenarians (23/299) without statistical significance (p = 0.52).

Observed mortality did not significantly differ between septua- and octogenarians even though a significantly different mortality was predicted by the logistic EuroSCORE.

The calibration of the univariate model including RIFLE stages was good (p = 1.0). The univariate odds ratios for 30-day mortality by RIFLE category were as follows (no AKI as reference): risk (1.05; 95% CI 0.35 to 3.08, p > 0.05); injury (2.99; 95% CI 1.1 to 8.2; p = 0.007) and failure (13.3; 95% CI 4.5 to 39.9; p < 0.001).

ROC analysis showed a fair discriminatory power (AUC = 0.69; SE 0.05; 95% CI 0.58 to 0.80). Considering the prevalence of mortality of 6.8% (41/598) in our sample, the positive predictive value (PPV) for death within 30 postoperative days was 15.7% (95% CI 10.1 to 23.5) and the negative predictive value (NPV) 95.5% (95% CI 93.1 to 97.1) using combined RIFLE stages 2 (injury) and 3 (failure) as "positive" test result and stages 0 (no AKI) and 1 (risk) as "negative" test result.

Greater degrees of AKI were also associated with prolonged stays at the intensive care unit (ICU) and at the hospital irrespective age (Table 5). The largest aggravations were found between patients with kidney injury and failure.

**Table 5:** ICU- and hospital stay at different AKI stages

Median ICU-stay [days]			
RIFLE stage	Septuagenarians n = 299	Octogenarians n = 299	p-value
No AKI	1 (1; 2)	1 (1; 3)	
Risk	2 (1; 3)	2 (1; 3)	
Injury	5 (2; 8)	4 (1; 7)	
Failure	14 (7; 30)	10 (3; 20)	0.32^A^

Median hospital stay [days]			
No AKI	11 (10; 14)	11 (9; 15)	
Risk	12 (10; 15)	13 (11; 17)	
Injury	14 (11; 19)	14 (11; 22)	
Failure	30 (17; 35)	23 (17; 26)	0.33^A^

^A ^- P-value is exemplarily shown. The differences between both age groups at other RIFLE stages also were not significant.

## Discussion

The number of patients older than 70 years referred for cardiac surgery has been steadily increasing in the recent years. This in part reflects improvements in life expectancy with a resultant ageing population [15]. Some studies have shown that survival and frequency of complications after cardiac surgery were comparable between septua- and octogenarians [16,17]. Other studies demonstrated increased mortality and morbidity in octogenarians compared to younger patients [18,19]. Preoperative renal dysfunction was identified as one of the most important risk factors for postoperative mortality. However, to our knowledge, there have been no studies published, which examined the incidence of postoperative AKI in patients ≥80 years compared to septuagenarians, who present a very large proportion of cardiac surgery patients in Germany.

Younger patients (18 to 69 years), which underwent CABG, valve and combined CABG and valve operations at the same time at our institution, not only had lower 30-day mortality (2.5%), but also a lower incidence of RRT after surgery (3.1%). In addition, the incidence of AKI according to RIFLE stages "injury" (10.5%) and "failure" (3.1%) was much lower compared to our sample of septua- and octogenarians. Even the number of patients at "risk" was lower 18.7%) reflecting that 67% of all patients had no AKI. These data emphasize the importance of AKI in an older cardiac surgery population and should increase the awareness of surgeons, but also of those, who do primary diagnostic procedures and initiate the referral for surgery.

As shown in a recent report [20], we confirm in our study that the RIFLE classification provides useful accurate risk assessment for 30-day mortality, in particular when patients are classified into the appropriate stage rather than AKI alone. Our results also are in agreement with the studies by Kuitunen and Ricci [21,22], which clearly demonstrated a stepwise increase in risk for death with each AKI stage from risk to failure in several patient populations. Our reported 30-day mortality rates at different AKI stages are, although restricted to patients ≥ 70 years, comparable to those published recently by the Northern New England Cardiovascular Disease Study Group [20].

Our study extends findings of prior studies by supporting that octogenarians do not develop postoperative AKI more frequently, when compared to a gender and surgical procedure matched cohort of septuagenarians [16]. However, approximately every fifth patient in both age groups developed kidney injury or failure and experienced increased risk for death. In addition, a large proportion of patients were at risk for AKI after surgery. Keeping the elevated mortality in mind, it remains essential to identify patients with increased risk for AKI with an appropriate risk score such as RIFLE or AKIN (Acute Kidney Injury Network) perioperatively [23].

Though octogenarians presented in our study with worse SCr and EuroSCORE-predicted mortality, they had a similar outcome as our septuagenarians. Optimal management, good preventive strategies, correct indications and a more frequent use of percutaneous techniques such as percutaneous coronary intervention (PCI) and transcatheter aortic valve implantation (TAVI) may help to improve the quality of life in octogenarians and also to circumvent cardiac surgery associated complications in those at high and very high risk.

There are limitations to consider in our analysis. We did not use urine output to aid in classifying postoperative renal function because all patients received diuretics at varying doses at the ICU and frequently before surgery and this would make urine output a less reliable indicator of renal function. Second, our study sample comprised only 598 patients, which is, compared to other studies, e.g. [11,20,24] rather low and may explain the only fair discriminatory performance of our ROC analysis. We also could not provide data on long-term mortality because they were not monitored at our center.

## Conclusion

In conclusion, the incidence of AKI defined with RIFLE criteria did not differ between septua- and octogenarians, but a significant proportion of patients in both groups developed kidney injury or was at risk for. The RIFLE criteria allow estimation of postoperative kidney dysfunction at advanced age. Greater degrees of postoperative AKI are associated with increased risk for death and prolonged ICU and hospital stay. Thus, octogenarians can be operated at acceptable risk, but careful preoperative selection for comorbidities remains essential in this patients.

## Competing interests

The authors declare that they have no competing interests.

## Authors' contributions

All authors read and approved the final manuscript.

MR: study design, data analysis, writing manuscript, revising the manuscript; TP: study design, data interpretation, helping drafting the manuscript; AH: study design, data interpretation, helping drafting the manuscript; CS: study design, correction of the manuscript; CD: study design, data collection and analysis, data interpretation, helping drafting the manuscript, revising the manuscript.

## Pre-publication history

The pre-publication history for this paper can be accessed here:

http://www.biomedcentral.com/1471-2261/11/52/prepub
